# Sulphur and Charcoal in Cholera

**Published:** 1849-08

**Authors:** 


*Sulphur and Charcoal in Cholera.*—Considerable public interest was awakened, a short time since, by the announcement of a supposed specific for Cholera, in a combination of charcoal and sulphur, administered in small doses, repeated at short intervals. Dr. Wm. B. Herrick, Prof. of Anatomy in the Rush Medical College, published a communication in the Chicago daily papers, expressing his confidence in the prophylactic and remedial virtues of this combination, attributing the merit of the suggestion to Dr. J. H. Bird, a young and talented practitioner at Chicago. Its supposed therepeutical value is based on the belief that epidemic cholera is produced by the presence, in the atmosphere, of a deleterious substance, called *Ozone*, to which the attention of the Profession has been directed, by Prof. Schonbein, of Germany. With reference to the composition of this substance, the reader is referred to two selected articles contained in the eclectic department of the present number of this Journal. Facts have been observed which go to show that there may possibly exist a causative connection between epidemic Influenza and the presence of *Ozone* in the atmosphere; and it has been remarked that Influenza so frequently has preceded epidemic cholera, as to lead to the supposition that there may exist some connection between the two diseases, as respects their causation. The suggestion that sulphur and charcoal may form a specific remedy for cholera, was made by Dr. Bird, as a chemical deduction, assuming that the foregoing postulates are correct. It must be at once apparent, that the data are, as yet, altogether hypothetical for much *a priori* confidence in the imagined discovery. The relations of *Ozone* to Influenza, and of the latter affection to Cholera, are suppositions, not facts; and, even if these relations were established, the efficacy of the proposed remedy would still remain to be determined. It is evident that if there exist a chemical antidote to the unknown poison which produces cholera, its operation must be chiefly prophylactic, since most of the characteristic phenomena of the disease, and its danger, are due to the blood-lesions occasioned by the serous effusion into the intestinal canal. How are these to be recovered from, even if we were fortunate enough to possess means for neutralizing the morbid conditions which precede them! In other words, the advantage of such a specific would consist in *prevention* rather than *cure*, and our means of prevention are now perhaps nearly all that could be desired.

It should be stated, however, that Drs. Herrick and Bird have prescribed the sulphur and charcoal remedy, to some extent, and it has appeared to them to exert a striking effect in relieving the premonitory symptoms of cholera, and producing marked results in some instances in which the disease has become confirmed. Their experience, however, in so far as appears from Dr. Herrick's publications in the Chicago papers, and the North Western Medical Journal, must be considered to be too limited to possess much importance as substantiating the claims of the remedy; and we are not aware that additional facts, with reference to this point, have been, as yet, contributed.

Drs. Herrick and Bird are entitled to praise for their zeal and enthusiasm, and for their philantropic desire to communicate, as speedily and widely as possible, the discovery supposed to have been made; but we fear we have yet to wait for a specific antidote to this fearful disease, and that we must be content to apply to its prophylaxis and treatment, general principles of Hygiene, Pathology, and Therapeutics.

